# Microbiome assembly statistics toward ecosystem-scale insights, forecasting, and management

**DOI:** 10.1093/ismejo/wrag085

**Published:** 2026-04-10

**Authors:** Hirokazu Toju, Kenta Suzuki, Martina Sánchez-Pinillos, Genta Shima, Takuya Kageyama, Ibuki Hayashi, Mikihito Noguchi, Hiroaki Fujita, Yoshiyuki Goto, Shinji Nakaoka, Masayuki Ushio, Yasunori Ichihashi, W Florian Fricke, Kenji Mizumoto, Lena Takayasu, Wataru Suda, Misako Takayasu, Masato Yamamichi, Wolfram Weckwerth

**Affiliations:** Laboratory of Ecosystems and Coevolution, Graduate School of Biostudies, Kyoto University, Kyoto 606-8501, Japan; Center for Living Systems Information Science (CeLiSIS), Graduate School of Biostudies, Kyoto University, Kyoto 606-8501, Japan; Integrated Bioresource Information Division, BioResource Research Center, RIKEN, Tsukuba, Ibaraki 305-0074, Japan; Institute for Multidisciplinary Sciences, Yokohama National University, Yokohama, Kanagawa 240-8501, Japan; Centre for Forest Research, Université du Québec à Montréal, H3C 3P8 Montreal, Canada; Laboratory of Ecosystems and Coevolution, Graduate School of Biostudies, Kyoto University, Kyoto 606-8501, Japan; Graduate School of Science, Kyoto University, Kyoto 606-8502, Japan; Laboratory of Ecosystems and Coevolution, Graduate School of Biostudies, Kyoto University, Kyoto 606-8501, Japan; Graduate School of Science, Kyoto University, Kyoto 606-8502, Japan; Laboratory of Ecosystems and Coevolution, Graduate School of Biostudies, Kyoto University, Kyoto 606-8501, Japan; Center for Living Systems Information Science (CeLiSIS), Graduate School of Biostudies, Kyoto University, Kyoto 606-8501, Japan; Project for Host-Microbial Interactions in Symbiosis and Pathogenesis, Division of Molecular Immunology, Medical Mycology Research Center, Chiba University, Chiba 260-8673, Japan; Division of Pandemic and Post-Disaster Infectious Diseases, Research Institute of Disaster Medicine, Chiba University, Chiba 260-8673, Japan; Division of Infectious Disease Vaccine R&D, Research Institute of Disaster Medicine, Chiba University, Chiba 260-8673, Japan; Chiba University Synergy Institute for Futuristic Mucosal Vaccine Research and Development (cSIMVa), Chiba University, Chiba 260-8673, Japan; Faculty of Advanced Life Science, Hokkaido University, Sapporo 060-0810, Japan; Department of Ocean Science, The Hong Kong University of Science and Technology, Clear Water Bay, Kowloon, Hong Kong SAR, China; Center for Sustainable Resource Science, RIKEN, 3-1-1 Koyadai, Tsukuba, Ibaraki, 305-0074, Japan; Department of Microbiome Research & Applied Bioinformatics, University of Hohenheim, 70599 Stuttgart, Germany; Graduate School of Advanced Integrated Studies in Human Survivability, Kyoto University, 1, Yoshida-Nakaadachi-Cho, Kyoto, 606-8306, Japan; Meinig School of Biomedical Engineering, Cornell University, Ithaca, NY 14853, United States; Center for Integrative Medical Sciences, RIKEN, 1-7-22 Suehiro-Cho Tsurumi-Ku, Yokohama, Kanagawa, 230-0045, Japan; Center for Integrative Medical Sciences, RIKEN, 1-7-22 Suehiro-Cho Tsurumi-Ku, Yokohama, Kanagawa, 230-0045, Japan; School of Computing, Institute of Science Tokyo, 4259, Nagatsuta-cho, Midori-ku, Yokohama, 226-8503, Japan; Institute for Multidisciplinary Sciences, Yokohama National University, Yokohama, Kanagawa 240-8501, Japan; Center for Frontier Research, National Institute of Genetics, 1111 Yata, Mishima, Shizuoka 411-8540, Japan; Genetics Program, Graduate Institute for Advanced Studies, SOKENDAI (The Graduate University for Advanced Studies), 1111 Yata, Mishima, Shizuoka 411-8540, Japan; Department of International Health and Medical Anthropology, Institute of Tropical Medicine, Nagasaki University, 1-12-4 Sakamoto, Nagasaki 852-8523, Japan; School of the Environment, The University of Queensland, St. Lucia, Brisbane, Queensland 4072, Australia; Department of Ecosystem Studies, Graduate School of Agricultural and Life Sciences, The University of Tokyo, 1-1-1 Yayoi, Bunkyo-ku, Tokyo 113-8657, Japan; Division Molecular Systems Biology, Department of Ecogenomics and Systems Biology, University of Vienna, Djerassiplatz 1, 1030 Vienna, Austria; Vienna Metabolomics Center (VIME), University of Vienna, Djerassiplatz 1, 1030 Vienna, Austria

**Keywords:** alternative stable states, attractors, community dynamics, competition, ecosystem functions, multistability, priority effects, regime shifts, stability

## Abstract

Microbiomes are increasingly recognized as key to addressing global challenges in health and sustainability, as they can provide emergent biological functions unattainable with single microbial species. However, microbial communities occasionally undergo abrupt shifts in species composition despite their intrinsic steadiness, making it difficult to maintain highly functional microbiome states. Here, we outline emerging statistical frameworks that integrate ecological stability theory with empirical analyses of microbiome structure and function. Approaches inspired by the concept of “stability landscapes” now enable inference of how the relationship between community structure and assembly potential changes along environmental gradients. Such empirical analyses offer bird’s-eye perspectives for maintaining or restoring community states with desirable microbiome functions. Moreover, identifying the attractors of microbiome dynamics facilitates forecasting of abrupt transitions into dysfunctional states (i.e. dysbiosis). Bridging classic ecological theory and empirical microbiome analyses will deepen our understanding of the principles governing species-rich community assembly, expanding the scope of microbiome-based solutions across medical, industrial, agricultural, and environmental sciences.

## Introduction

Biological communities are formed by diverse species differing in gene repertoires [1–3]. Owing to the diversity of constituent species and their genomes, communities can vary in ecosystem-scale functional profiles [4–6] despite conservatism in system-specific basic properties [7, 8]. Bacterial communities in the human gut, for example, impact host health/disease status (e.g. obesity, type-II diabetes, neurological disorder, and immune responses to pathogens) depending on their taxonomic compositions [1, 5, 9]. Likewise, the community structure of plant-associated bacteria and fungi determines crop growth and yield in agriculture [4, 6]. In terrestrial and aquatic ecosystems, the ecosystem-level emission/sequestration rate of greenhouse gas depends on the community structure of bacteria, archaea, and fungi involved in carbon/nitrogen cycles [2, 10]. Thus, cataloging the structure and functions of microbiomes is an essential step for making the most of microbial functions in diverse fields of applied sciences [11–16].

**Figure 1:**
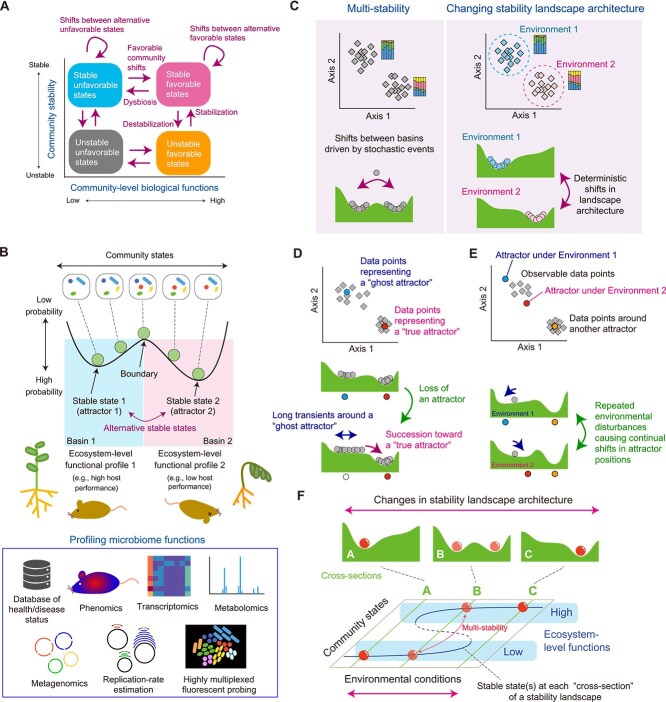
Microbiome structure, stability, and functions. (A) Functions and stability as key properties of biological communities. (B) Classic schema of stability landscapes. Community states (community memberships; horizontal axis) can differ in their potential (vertical axes). On the illustrated stability landscape, basins of attraction are split by a boundary representing an unstable state. The bottoms of basins represent stable states (attractors). Communities belonging to different basins may differ substantially in their ecosystem-level functions, which can be profiled with diverse omics approaches. For example, phenotyping technologies [103, 104], transcriptomics [102], and metabolomics [107, 108] allow high-throughput profiling of the health/disease status of host animals/plants. Metagenome-based analyses of replication rates [113] and multiplex imaging with >1000 fluorescent probes [112] will provide novel insights into the physiological roles of symbionts within hosts’ tissue. (C) Two scenarios behind microbiome types. If multiple basins exist within a stability landscape, community states would be grouped into clusters even under identical environmental conditions (multi-stability; left). If the architecture of stability landscapes changes due to shifts in environmental conditions, clusters within the state space would represent environmental dependence of community states (deterministic shifts in stability landscape architecture: right). Real ecological communities can be driven by both scenarios. (D) Long transients on stability landscapes. Community structural change may slow down in a flat part of a stability landscape, causing clusters of community compositions around a “ghost attractor” [58] (representing long transients; Box 1) and a “true attractor.” In such cases, statistical analyses of data-point distribution based on the classic multi-stability framework (B) may fail to capture ecological processes behind community dynamics. (E) Disturbances and transient states. Communities may remain in transient states for extended periods due to repeated shifts in environmental conditions. (F) Stability landscape dynamics. The number and position of basins can change along gradients of environmental conditions. Therefore, drastic shifts in community states and related ecosystem-level functions can occur at certain points along such environmental gradients.

Nonetheless, the prospects of optimizing microbiomes for medical, agricultural, and environmental issues [11, 12, 14, 17, 18] are often hampered by the complexity of microbial community dynamics [19–23]. Even if microbial species sets with desirable functions are obtained in laboratory co-culture experiments [15, 24], the species compositions of the designed microbiomes may be hard to maintain for long periods of time in real ecosystems [25–27]. In other words, transitions to community structure with unfavorable functions (i.e. “dysbiosis”) can occur in the dynamics of animal-/plant-associated microbiomes, as well as those of environmental microbiomes [5, 12, 20, 28, 29]. In deepening our knowledge of such transitions between alternative stable states [30–32] (Fig. 1A), community-scale stability, which is represented by resilience, resistance [33–35], and various early warning signals [36–39], needs to be considered as a critical property of microbiomes [25, 40–44]. In ecology, community stability has been discussed by mainly targeting macro-organismal systems such as animal and plant communities [45, 46]. Given the growing availability of large-scale data on microbial communities, developing integrative frameworks to evaluate community stability is a crucial first step for managing ecosystem-scale functions of microbiomes.

**Box 1. Glossary.**

**Assembly landscapes** refer to a statistical reconstruction of how likely different community states (species assemblages) are observed within the state space of possible species combinations.

**Alternative stable states** refer to a concept in which an ecosystem has multiple stable compositions (or memberships) of species under the same set of environmental conditions.

**Community stability** refers to the ability of an ecological community to maintain its structure. Community stability measures frequently discussed in theoretical ecology are ecological resilience (the ability of a system to absorb perturbations to community states), engineering resilience (the speed at which a system returns to its original state after a perturbation), and resistance (the ability of a system to remain unchanged after a disturbance).

**Entropy** is a metric of uncertainty, randomness, or information content when it is referred to in the context of information theory (i.e. Shannon entropy).

**Ghost attractors** are defined as community states in which equilibria once existed but were lost following reorganization of the stability landscape. Communities may remain in the vicinity of ghost attractors for extended periods before transitioning to (true) attractors.

**Hysteresis** refers to the dependence of systems’ states on their history. The concept is often mentioned to illustrate complex dynamics in which the path of the recovery of a system is different from the path of its collapse.

**Panomics** refers to an extension of multi-omics approaches that integrates all relevant omics information to gain comprehensive understanding of biological systems, rather than focusing on a limited set of molecular layers.

**Stability landscapes** refer to conceptual representations of the relationship between the structure and stability of biological communities.

**Transient states** refer to nonequilibrium phases in which a system remains for a limited but potentially significant period of time.

We here outline the concepts of “multi-stability” and “stability landscapes” [30–33, 35], which have motivated the development of general frameworks for understanding the relationship among community structure, stability, and functions (Fig. 1B). We then introduce the ways by which the risk of drastic community-scale changes (e.g. dysbiosis) can be empirically evaluated either by using the framework of statistical physics or by tracking trajectories of microbiome dynamics. Lastly, we discuss how we can forecast transitions between microbiome states based on data-driven approaches. Overall, this paper illustrates how emerging statistical platforms accelerate the feedback between the theory and empirical studies of species assembly, providing general and practical bases for taming the enormous complexity of microbiome functional dynamics.

## Classic stability landscape concept

Microbial communities (microbiomes) are structured through complex combinations of deterministic and stochastic ecological processes [31, 39, 47, 48]. Therefore, microbiome datasets yield system-specific unique distributions of samples within the state space of possible species compositions [4, 49, 50]. In a seminal work on human gut microbiomes, for example, microbiome samples were inferred to cluster into several types (enterotypes), which might differ in physiological impacts on host individuals [49]. Likewise, in agroecosystems, soil microbiome structure can be represented by several types that substantially differ in their associations with crop disease status [51]. Such microbiome types have been examined based on clustering approaches for detecting heterogeneous distributions of data points [51, 52] (Fig. 1C), although the interpretation of the clustered patterns is often controversial in terms of the presence of intermediate data points connecting recognizable clusters [53–55].

Accumulating knowledge of microbiome types is now being integrated into discussions based on the “multi-stability” perspective of ecological communities [30, 31, 35, 43, 56]. In classic theory, community states are expected to converge toward stable states. If multiple stable states exist (i.e. if multi-stability underlies community assembly), drastic changes in ecosystem-level functions can occur due to transitions between community compositional states after large stochastic changes in community structure [22, 30, 35, 57]. Such ecological processes have been intuitively grasped with the concept of “stability landscapes” [30, 31, 35]. In the classic ecological concept of stability landscapes, the current community structure is represented by a ball rolling within a valley (i.e. a basin of attraction represented by a stable state) or crossing a hilltop (i.e. boundary between basins) toward alternative stable states [30–32] (Fig. 1B).

The multi-stability interpretation of clustered data points (community states) has recently been reconsidered in light of the possibility of long transient states in community dynamics [58, 59]. In this extended framework, some observed clusters may represent long transients rather than true stable states. For example, a system may require a long period to traverse its entire state space, particularly in the presence of saddle points or “ghost attractors” [58, 59] (Fig. 1D). Theoretical studies predict that long transients are more likely in systems with frequent stochastic events and high dimensionality [58, 59] (i.e. communities with many species). It is also important to recognize that transient dynamics may be empirically indistinguishable from stable states, and they may represent a fundamental aspect of community behavior. In real ecosystems, persistent demographic noise and ongoing environmental disturbances can keep communities in transient states for extended periods, making them appear as if they were stable (Fig. 1E). Although pioneering studies have deepened our understanding of transient dynamics [58, 59], robust methods for distinguishing transient states from stable ones are still lacking. Therefore, the concept of multi-stability in ecological dynamics should be applied with an awareness that the development of statistical frameworks capable of disentangling transient and converging phenomena remains an ongoing challenge.

When we apply currently available statistical approaches, we also need to keep in mind that clustering analyses alone do not provide explicit insights into underlying ecological processes. Even if multiple clusters of community compositions are detected, they may merely represent community structures under different environmental conditions [30] (Fig. 1C right). In other words, the presence of community structural types may be attributed to differences in the positions of globally stable points under different environmental conditions (Fig. 1C right), but not to multi-stability (i.e. the presence of multiple attractors) under identical environmental conditions (Fig. 1C left).

In considering how the relationship between community structure and stability depends on environmental conditions (e.g. pH and temperature), we can conceptualize changes in the topography of a stability landscape as “cross-sections” along the axis of an environmental variable [30, 31, 35, 60] (Fig. 1F). In theory, the number and position of stable states (i.e. bottoms of valleys within stability landscapes) can drastically change depending on background environments [31, 35, 60]. Therefore, statistical frameworks for modeling the flexibility of stability landscapes are expected to advance microbiome research beyond simple clustering approaches of community compositions.

## Empirical analyses of assembly landscapes

**Figure 2:**
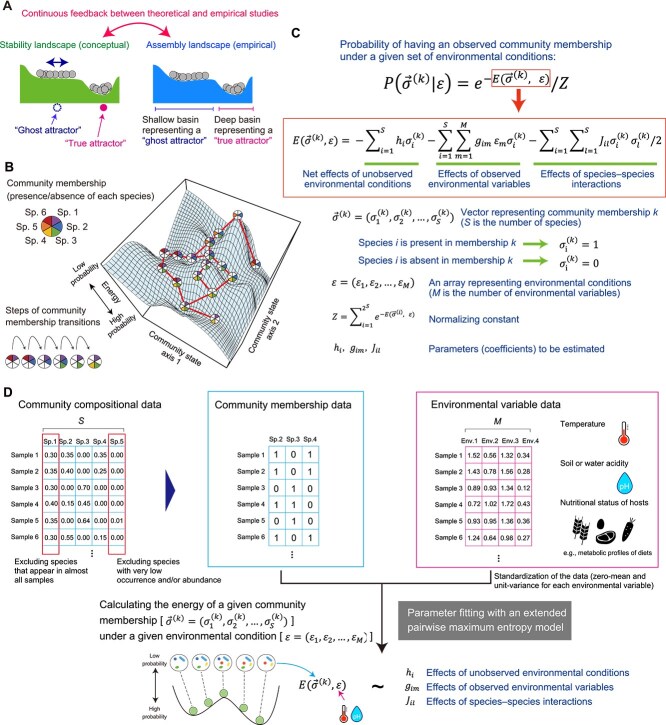
Energy landscape analysis. (A) Stability and assembly landscapes. Stability landscapes illustrate the conceptual relationship between the structure and stability of communities, whereas assembly landscapes inferred from empirical data describe the statistical relationship between community states and probabilities (see Glossary). (B) Schema of an energy landscape. Changes in community membership are modeled as steps within an “assembly graph.” (C) Statistical model. The probability of yielding the observed memberships of species is modeled as a function of “energy,” which is expressed as a linear combination of environmental and species-interaction effects on community structure [60]. In the energy landscape formulation, ${h}_i$ denotes the implicit impacts of the environment on the occurrence of species *i*, ${g}_{im}$ represents the dependence of species *i*’s occurrence on explicit environmental variable *m*, and ${J}_{il}$ represents potential interactions between species *i* and *l*. These parameters were estimated by fitting an extended pairwise maximum entropy model, using stochastic approximation with a persistent Markov chain Monte Carlo sampler to approximate otherwise intractable model expectations. Once the parameters (model coefficients) are fit, the energy of a given community state under each set of environmental conditions is estimated. Thus, energy landscape analysis provides a basis for quantitatively understanding how assembly landscape architecture changes along environmental gradients. (D) Flow of energy landscape analysis. For a first step, a data matrix describing community states is prepared. The matrix may be provided by amplicon sequencing (e.g. 16S rRNA gene amplicon sequencing of prokaryotes) or metagenomic sequencing. The input matrix is converted into a binary (membership) format [51] (see Box 2 for details). The binary matrix describing community states and a matrix showing the environmental conditions of corresponding samples are then subjected to statistical parameter fitting [38, 60]. The obtained coefficients representing the impacts of environmental conditions and those representing species-to-species interactions on community states are used to calculate the energy of a given community state (membership) in each set of environmental variable values.

We hereafter introduce statistical frameworks for inferring the dynamic nature of stability landscapes. Reconstructing the architecture of stability landscapes from empirical data is generally a challenging task. As discussed above, ghost attractors are often indistinguishable from true attractors (Fig. 1D and E). Given these potential pitfalls, we refer to landscapes reconstructed from community assembly (assemblage) data as “assembly landscapes.” The topography of assembly landscapes is expected to reflect key properties of the underlying stability landscapes. Deep basins inferred in an assembly landscape analysis should correspond to those in stability landscapes, whereas shallow basins may represent ghost attractors (Fig. 2A). In other words, although perfect correspondence between theoretical landscapes and empirical observations based on finite and noisy data is unattainable, practical statistical frameworks for overviewing ecological community processes provide an essential starting point for bridging theoretical and empirical research [36, 38, 60, 61].

Although statistical approaches to assembly landscapes are inspired by the classic stability landscape concept, the estimated assembly landscapes themselves can be interpreted within alternative theoretical frameworks. Basins identified from empirical data do not necessarily represent equilibria (point attractors); rather, they may correspond to other types of attractors. For example, one basin on an assembly landscape may capture host–parasite oscillatory dynamics (limit cycles) involving species sets A and B, whereas another basin may represent distinct dynamics involving species sets C and D. Assembly landscapes simply depict the probability projections of the outcomes of ecological processes. Because the type of attractors cannot be known *a priori*, assembly landscape topography should be interpreted flexibly with consideration of possible alternative attractor types.

A statistical approach for systematically analyzing the dependency of assembly landscape architecture on background environments has been recently proposed in light of statistical physics, which provides general platforms for evaluating probability distributions of systems’ states. In the framework of “energy landscape analysis” [60–64], community ecological processes are interpreted as shifts between nodes within an “assembly graph” [65–67], which represents a network of community membership (Box 2: Fig. 2B). The probability of observing a given community state (community membership) is then expressed as a decreasing function of an “energy” metric. Specifically, the community energy metric is statistically defined as a linear combination representing the impacts of environmental variables and species interactions on the probability of yielding observed community memberships [60, 61] (Fig. 2C and D). By incorporating environmental variables such as temperature and acidity of the aquatic/soil environments and nutritional status of host animals/plants, the statistical framework allows us to infer how the landscape architecture changes depending on background environmental conditions. Even if data on environmental conditions are unavailable, implicit variables representing the net effects of unobserved environmental factors can be included in the statistical analysis [60]. In the fitting of the statistical parameters with an extended pairwise maximum entropy model [60, 68] (Fig. 2C and D), the term “energy” is employed to express how often a given state is observed (or unobserved), following analogous applications of statistical-physics formulations in ecology [68] and brain science [63]: this usage does not correspond to any physical form of energy. In this sense, this terminology is conceptually comparable to that of “potential” in other mathematical approaches on community multi-stability [32, 35, 43]. On the inferred energy landscape, valleys represent frequently observed community memberships, whereas hilltops represent boundaries splitting the valleys (basins) (Fig. 2B). In a benchmark analysis with generalized Lotka–Volterra (gLV) competition equations, energy landscape analysis identified discrete community states to which convergence from randomly generated initial states occurred through simulations [60]. More generally, energy landscape analysis provides a model-agnostic way to infer basins and boundaries from empirical community data while remaining compatible with interpretations based on deterministic dynamics such as gLV or consumer–resource models. Although further theoretical developments are warranted, energy landscape analysis provides a practical framework for characterizing the architecture of assembly landscapes.

**Box 2. Applying energy landscape analysis.**

In applying energy landscape analysis for overviewing community assembly (Fig. 2), there are some points to be considered carefully. First, to calculate the probability of multi-dimensional community states, large numbers of microbiome samples need to be prepared, as is often the case for statistical analyses of ecological communities [26, 47, 86]. Although the minimal required number of microbial communities can vary depending on the complexity of the state space (e.g. the number of attractors), at least 100 microbiome samples are generally required to obtain reliable results [36, 60]. This requirement is not specific to energy landscape analysis: unless the target system converges immediately to a deterministic equilibrium without stochastic fluctuations, ≥100 samples are necessary regardless of the statistical frameworks employed. Given the input data requirements, the application of energy landscape analysis will be expanded from large-scale project datasets (e.g. human microbiome projects) to specific microbiome studies with well-conceived sampling designs (e.g. ≥200 soil microbiome samples spanning the full range of a pH gradient). Second, analyses based on assembly graphs (Fig. 2B) currently require binarization of input data. This binarization step inevitably leads to a loss of information from original quantitative data, potentially limiting the power of energy landscape analysis. Despite this methodological limitation, the early warning signals defined by energy landscape analysis (Fig. 5A) significantly forecasted future large shifts in community compositions in an experimental microbiome study [36]. The improvement of forecasting skill will be achieved by integrating pipelines of raw-data processing such as the natural language processing of the latent Dirichlet allocation model for quantitative microbiome data [62]. Third, processing big data requires substantial computational power, as is always the case for statistical analyses of species-rich microbiomes. In soil microbiomes, for example, the number of detected operational taxonomic units (OTUs) can exceed 10^4^, making even basic statistical approaches (e.g. permutational analysis of variance) computationally intensive [73]. Therefore, when analyzing data with substantial species diversity, taxonomic membership (genus-, family-, or order-level membership), instead of species-level membership, may be used to reduce computational burden. Species or taxa that occur in almost all samples can be deleted from the input data because, while increasing computational load, their presence in the data matrix contributes little to among-sample variability in community membership. Likewise, species or taxa occurring in only a few samples may be cut unless there is concrete prior knowledge of the overwhelming impacts of the rare species/taxa on the entire microbiomes. To gain reliable inferences of assembly landscape architecture, it is essential to verify the consistency of results across multiple criteria (occurrence and abundance thresholds) used to summarize input data [51]. In terms of minimizing information loss through binarization, optimal thresholds for occurrence and abundance can be selected by comparing pairwise dissimilarity matrices derived from the original quantitative data (e.g. Bray–Curtis beta-diversity) and those from the binarized data (e.g. Jaccard beta-diversity), and identifying the thresholds that maximize the correlation between the two matrices. The packages for energy landscape analysis are available for Mathematica (https://community.wolfram.com/groups/-/m/t/2358581), MATLAB (https://github.com/tkEzaki/energy-landscape-analysis), R (https://github.com/kecosz/rELA), and Python (https://github.com/KaiyangZ96/Energy_landscape;  https://github.com/okumakito/elapy;  https://github.com/sotarotakano/pyELA) languages.

**Figure 3:**
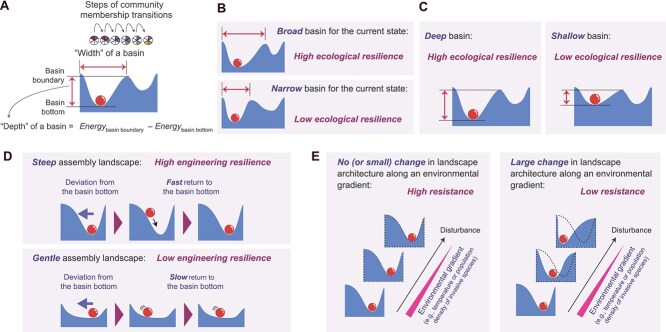
Community resilience and resistance illustrated on assembly landscapes. (A) Topographic features of assembly landscapes. (B) Ecological resilience and basin width. A community located within a broader basin is less likely to shift into an alternative basin following perturbations to its state [32, 33, 70]. (C) Ecological resilience and basin depth. The relative depth of a basin bottom to the basin boundary represents the amount of “effort” required for a community state to shift into an alternative basin, providing an alternative measure of ecological resilience [35]. (D) Engineering resilience. When the state of a community is perturbed, the time required to return to the basin bottom depends on the local geometry of the basin (i.e. balance between width and depth) [35, 39]. In a highly resilient system, community states return quickly to the basin bottom. (E) Resistance. Ecological resistance can be defined in terms of a system’s responses to environmental disturbances. Specifically, in a resistant system, communities are expected to withstand disturbance imposed by external environmental changes. From the perspective of empirical research using energy landscape analysis, the resistance of a system is evaluated based on the degree to which basin architecture remains unchanged along environmental gradients.

Overall, energy landscape analysis enables the statistical description of how assembly landscape topography changes along environmental gradients, grounded in the maximum entropy criterion. Using the parameter sets estimated in the statistical framework, we can quantitatively evaluate the topography of basins [60, 69] (Fig. 3A). Thus, energy landscape analysis helps us discuss the resilience and resistance of target systems [33, 35, 39, 60]. Among the two major concepts of resilience, ecological resilience, defined as the capacity of a system to absorb perturbations to community states [33, 70], can be evaluated by the “width” or “depth” of the basin within which the current community state is located [35, 71] (Fig. 3B and C). The other major resilience concept, engineering resilience, is defined as the time required for a system to return to its original state after perturbation [32, 33, 39, 70]. On an assembly landscape, community states return more rapidly to basin bottoms after deviations when they reside within steep basins (i.e. high engineering resilience; Fig. 3D). Accordingly, basin geometry, as characterized by its depth–width structure, provides a basis for evaluating engineering resilience [35, 39]. In contrast to resilience, resistance is conceptually defined as the extent to which a system remains unchanged after a disturbance [33, 72]. When the effects of environmental disturbances (e.g. changes in temperature or acidity) on assembly landscape topography are statistically inferred from empirical data (Fig. 2), resistance can be interpreted as the steadiness of assembly landscapes *per se.* Accordingly, in the framework of the energy landscape analysis, the basin structure of a resistant system remains largely unchanged along broad ranges of environmental gradients (Fig. 3E).

## Hysteresis and microbiome control

Given that the number and position of attractors can change depending on environmental conditions [30, 31, 35], alternations of assembly landscape topography themselves deserve intensive statistical investigations. In other words, to develop comprehensive plans for managing microbiome structure and its related functions, we need to understand how assembly landscape architecture changes along the gradients of environmental variables.

**Figure 4:**
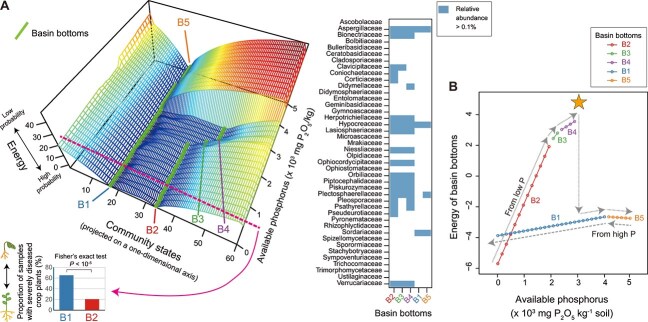
Environmental dependence of assembly landscape architecture. (A) Environmental gradients of assembly landscape architecture. Energy landscape analysis was applied to the fungal community dataset of 1664 cropland soil samples across the entire range of the Japan Archipelago [51, 73]. The multidimensional information of community states (community memberships) is projected onto a one-dimensional axis. Along the gradient of soil phosphorus concentrations, assembly (energy) landscape architecture at given environmental conditions (available soil phosphorus concentrations) is shown as cross-sections (center graph). The number of basins and the community memberships of basin bottoms change along the environmental gradient. Due to the substantial diversity of species in the data, the family-level taxonomic memberships were examined (right heatmap): families appearing in most (>90%) samples were excluded (see Box 2 for details). The bottoms of basins B1 and B5 have similar taxonomic memberships, whereas those of basins B2, B3, and B4 are grouped together. At a low phosphorus level (dashed line in the center graph), the inferred energy landscape exhibited a “bistable” architecture consisting of basins B1 and B2. An additional analysis using the sampling-point metadata [51] indicated that soil microbiome states located within basin B1 were more strongly associated with severe crop disease (percentage of diseased plants or disease severity index ≥80) than those located within basin B2 (left bar plot). Because the soil microbiome dataset comprised samples from diverse crop species, the crop with the largest number of data points (tomato) was selected for the disease severity analysis (basin B1, 295 metadata points; B2, 29 metadata points; the result of Fisher’s exact test is shown). (B) Hysteresis. At the minimal phosphorus level, the community state is positioned within basin B2, which has the lowest energy in the assembly landscape. As the phosphorus level increases along the gradient, the community state is expected to remain within its original basin without large stochastic fluctuations. With a further increase in phosphorus, the community state transitions sequentially from B2 to B3, and then to B4. Beyond a certain threshold along the environmental gradient (indicated by a star; ca. 3000 mg P_2_O_5_/kg), the original basin disappears, causing the community state to shift into alternative basin series (from B1 to B5). The community state follows a different trajectory when the environmental gradient changes in the reverse direction (from high to low phosphorus levels). This dependence of the community state on its historical pathway is referred to as hysteresis in physics. The vertical axis shows the range over which the energy values of basin bottoms vary, whereas that of (A) represents the full energy range.

Consider an example case in which the energy landscapes analysis is applied to a dataset comprising soil fungal communities along an environmental gradient [51, 73] (Fig. 4). At low soil phosphorus concentrations, microbiome states may be trapped within the basin with the minimal energy (B2 in Fig. 4) unless substantial demographic deviations occur. As the phosphorus level increases, the community membership of the basin bottom is shifted to some extent (B3 and then B4). As the environmental variable further increases to a certain threshold, the original basin may disappear, causing drastic shifts of community states into another basin with much lower taxonomic diversity (B1). From the aspect of directional environmental alternations, this microbiome process is irreversible. Specifically, when gradual environmental changes from high to low soil phosphorus concentrations occur, communities are expected to take a different route than they took in the previous shift from low to high phosphorus levels (Fig. 4B). Such dependence of the system’s states on their history [31, 35] (i.e. hysteresis) is central to managing microbiome structure and functions. In the cropland example, when phosphorus levels exceed the inferred threshold due to excessive fertilization, soil community states become “captured” by the low-taxonomic-diversity basin associated with severe crop disease (B1). Once this transition occurs, microbiome states cannot return to less diseased regimes (B2–B4) without substantial demographic fluctuations or community reconfiguration (e.g. massive introduction of specific microbial agents), even if phosphorus is decreased to its lowest level (Fig. 4). Thus, energy landscape analysis provides quantitative insights into the “points of no return” beyond which microbial ecosystems fall into dysfunctional regimes.

By applying the statistical framework for inferring assembly-landscape alternations along environmental gradients, we will gain integrated knowledge for taming the historical contingency [74] of microbiome dynamics [1, 75–77]. In this respect, the breadth of environmental ranges within which multi-stability exists can be used as a metric representing the strength of hysteresis, deepening our understanding of microbial ecological processes. Specifically, in systems with strong signs of hysteresis, resident community members may have great impacts on the proliferation of subsequent colonizers [75]. Such priority effects [74] may be particularly strong in soil or plant-root-associated fungal communities [78], in which resident fungal species can impede the colonization of other species by building physical barriers (hyphal structures) or producing inhibitory chemicals (antibiotics). Likewise, strong priority effects are expected to prevail in gut-associated microbiomes under the presence of microbes producing chemical compounds with negative or positive impacts on other microbes (e.g. short-chain fatty acids) or those working as preemptive consumers of essential metabolites. Indeed, in the infant gut, *Bifidobacterium bifidum* and *B. longum* subsp. *infantis* dominate as primary consumers of human milk oligosaccharides (HMOs), whereas their presence facilitates the colonization of *B. breve*, which has limited HMO-utilization ability and is therefore supported by HMO degradation products [75]. Changes in the availability of HMO degradant (e.g. fucose) through artificial nutrition would drastically change the landscape architecture of *Bifidobacterium* species assembly.

## Forecasting dysbiosis

Statistical analyses of assembly landscapes also provide a basis for forecasting abrupt shifts in microbiome structure and functions. In the classic schema of a ball rolling on a landscape (Fig. 1B), a community is expected to remain around the current state if it is located near the bottom of a basin. In contrast, stochastic events may cause a shift into a neighboring basin if the state of a community is located near a boundary between basins [45].

**Figure 5:**
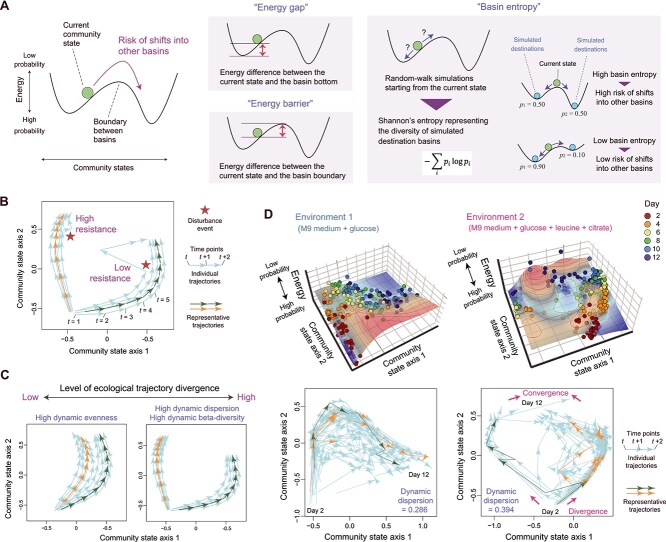
Temporal dynamics. (A) Anticipating drastic shifts. The risk of community state shifts between basins can be evaluated with indices defined based on energy landscape analysis. “Energy gap” and “energy barrier” are defined by the difference in the energy metric between the current state and the basin bottom, and between the current state and the basin boundary, respectively [36, 38]. “Basin entropy” (formerly referred to as “stable-state entropy” [36]) quantifies the diversity of potential outcomes of community assembly. Specifically, random-walk simulations of community assembly on the inferred landscape are performed by setting a target community state as a starting point. Each simulation is stopped when a community reaches a basin bottom. Across multiple simulation trials (e.g. 10 000 iterations), Shannon’s entropy representing the heterogeneity of destination basins is calculated as a metric of the risk of drastic community shifts. (B) Schema of ecological dynamic regimes. A sequence of arrows indicates community compositional shifts between time points for each replicate community. After merging time-series (ecological trajectory) data of multiple replicates (e.g. longitudinal data of multiple host individuals in gut microbiome research), “representative trajectories” characterizing the system’s dynamics are highlighted (Box 3). In the ecological dynamic regime framework [47, 81], a resistant community is defined as one whose structure remains unchanged after a disturbance event (e.g. antibiotic treatment). (C) Divergence of ecological trajectories. The level of trajectory divergence is quantified using the “dynamic dispersion,” “dynamic beta-diversity,” and “dynamic evenness” indices [47]. (D) Empirical analyses on ecological trajectories. Energy landscape analysis was applied to the dataset of a laboratory microbiome experiment [82] in which the changes in bacterial community states were monitored through six time points (from Day 2 to Day 12) with 48 replicates under each environmental (medium) condition (top). The statistical framework of ecological dynamic regimes was applied to the same time-series data to track the trajectories of community dynamics. Changes in community states between time points are shown as arrows for each of the 48 replicate microbiome samples. Clearer divergence into distinctive representative trajectories was observed early in the dynamics under a more complex medium condition (Environment 2; M9 minimal medium with glucose, leucine, and citrate). The analysis also suggested that convergence into similar community states occurred later in the community assembly under the environmental condition. The statistical framework was applied to a subset of the source dataset, comprising two of the eight medium conditions [82].

There are some metrics for evaluating the risk of drastic microbiome shifts in light of assembly landscape architecture (Fig. 5A). Because an assembly landscape represents how the probability of observing specific community states changes through community dynamics, its topography information can be used to evaluate the risk of drastic community-scale events (e.g. dysbiosis). Difference in energy between a community state (representing a microbiome sample) and the bottom of its basin is a prospective index (“energy gap”) [36, 38]. Likewise, energy difference between a community state and the lowest basin boundary around the state point would be used as a warning signal (“energy barrier”) [36]. Furthermore, random-walk simulations on the inferred assembly landscape allow us to evaluate the diversity of possible future states by calculating a Shannon entropy metric that represents uncertainty in destination basin bottoms (“basin entropy”) [36, 38]. Indeed, an application of energy landscape analysis to 110-time-point microbiome datasets has shown that a sudden and substantial reduction of taxonomic diversity of bacteria can be predicted to occur when the value of a warning signal (basin entropy) exceeds a certain limit [36]. Thus, once statistical parameters of target microbial ecosystems are obtained (Fig. 2D), drastic shifts in microbiome structure and functions can be anticipated for a given community state within the state space of microbiome dynamics.

To date, various types of “early warning signals” [37, 39, 79, 80] have been proposed to forecast the regime shifts [44, 45] of ecological communities (e.g. variance and autocorrelation in the population dynamics of species constituting a community). Compared with previously examined early warning signals, metrics based on the reconstruction of assembly landscapes are unique in that they can be extended to anticipate not only the risk of transitions, but also the potential paths and destinations of community state changes. In other words, the risk of microbiome shifts calculated by energy landscape metrics is interpreted, along with the assembly graphs representing transitions between basins via specific boundaries (Fig. 2B). Further improvements of the warning indices will help us quantify the potential of shifts into specific favorable or unfavorable states of microbiomes.

## Divergence of ecological trajectories

For further enhancing our knowledge of microbiome processes, it is crucial to perform detailed time-series analyses of community structural changes. In the statistical framework of ecological dynamic regimes [47, 81], an ecological trajectory is defined as a sequence of shifts in community structure across time points (Fig. 5B). The resistance of a replicate community is then defined as the degree to which the system remains unchanged after a drastic disturbance event (e.g. a typhoon or antibiotic treatment), quantified as dissimilarity in community structure before and after the disturbance [81]. The statistical framework can be further used to quantify the extent to which replicate communities diverge into “alternative trajectories” of community assembly based on the dynamic dispersion index and dynamic beta-diversity metrics [47, 81] (Box 3; Fig. 5C).

**Box 3. Ecological dynamic regime framework.**

According to the ecological dynamic regime framework [47, 81], the assembly landscape is generated from a multidimensional state space in which the basins of attraction are defined by groups of ecological trajectories (time series) following similar dynamical patterns (i.e. ecological dynamic regimes) under analogous environmental conditions. Within each dynamic regime, attractors can be associated with the regions densely occupied by ecological trajectories summarized in a set of representative trajectories (Fig. 5B). Ecological dynamic regime framework then allows us to quantify the degree to which the trajectories are dispersed based on “dynamic dispersion,” “dynamic beta-diversity,” and “dynamic evenness” metrics, as well as based on the divergence between two dynamic regimes or their changes over time using a dissimilarity metric [47] (Fig. 5C and D). Whereas energy landscape analysis examines how assembly landscape topography changes along environmental gradients (Fig. 4), ecological dynamic regime framework shows the overall pictures of ecological trajectories, which reflect both stochastic fluctuations and background environmental changes. Thus, the dynamic regime analysis provides a straightforward way for tracking community dynamics under specific environmental conditions and a way for using those dynamics as a reference to assess the divergence of trajectories caused by extreme stochastic events, planned anthropogenic interventions, or gradual environmental changes [47, 81]. In particular, the dynamic regime framework helps us track the recovery of microbiome structure after abrupt disturbance events (e.g. antibiotic treatments). The magnitude of disturbance events and communities’ responses to the disturbance can be quantified based on four metrics defined within the dynamic regimes (resistance, amplitude, recovery, and net direction) [81]. In applications to human gut microbiomes, for example, it is feasible to compare the resistance of gut microbiome structure against antibiotic treatments among host human individuals (Fig. 5B) or to evaluate the effects of different diets within each host individual. These analyses and metrics in the ecological dynamic regime framework can be implemented in the R package “ecoregime” [47, 81].

When time-series data of multiple replicate communities are available, such differentiation of ecological trajectories can be quantitatively evaluated. Laboratory experiments of bacterial communities have shown that species compositions can diverge along several alternative directions even when environmental conditions are strictly controlled across replicate experimental trials [43, 82, 83] (Fig. 5D). In a series of experiments with a highly replicated design, for example, a field-collected (forest soil) bacterial community was introduced into eight types of artificial media that varied in carbohydrate compositional complexity, with 48 replicates per condition [82]. Microbiome compositions were then tracked every 48 h for 12 days, yielding 2304 community samples (8 medium types × 48 replicates × 6 time points). Under most medium conditions, the 48 replicate communities clearly diverged into multiple alternative compositional trajectories. This pattern indicates that slight initial differences in community compositions, together with subsequent ecological drift, can cause divergence into multiple basins within an assembly landscape [74]. In other words, stochastic sampling effects (i.e. multinomial draws of bacterial cells from a source community) can cascade into discrete time-series patterns of community structure.

Application of the ecological dynamic regime analysis to this time-series dataset [82] further indicated that divergence into alternative trajectories was greater under complex than under simple medium conditions. For example, the dynamic dispersion index was higher in the media containing three carbon sources (glucose, leucine, and citrate) than in single-carbon-source media (glucose only) (Fig. 5D). Such comparative analyses within a quantitative statistical framework deepen our understanding of how niche complexity (e.g. the number of nutritional sources) determines the fundamental architectural properties of assembly landscapes (e.g. the number of basins). In addition to early-stage splitting dynamics, ecological dynamic regime analysis also identified sudden convergence of community states at later stages in the experimental microbiome assembly (Fig. 5D right). Overall, these insights into ecological trajectories advance our understanding of the pace and complexity of microbiome time-series processes [36, 47, 58], beyond simplified discussion assuming rapid convergence to stable states. For further comprehensive understanding, it will be important to examine whether the observed initial divergence reflects the presence of alternative transient states [74] (or alternative transient trajectories) that may ultimately converge on a true attractor [58, 59].

## Tracking complex dynamics

**Figure 6:**
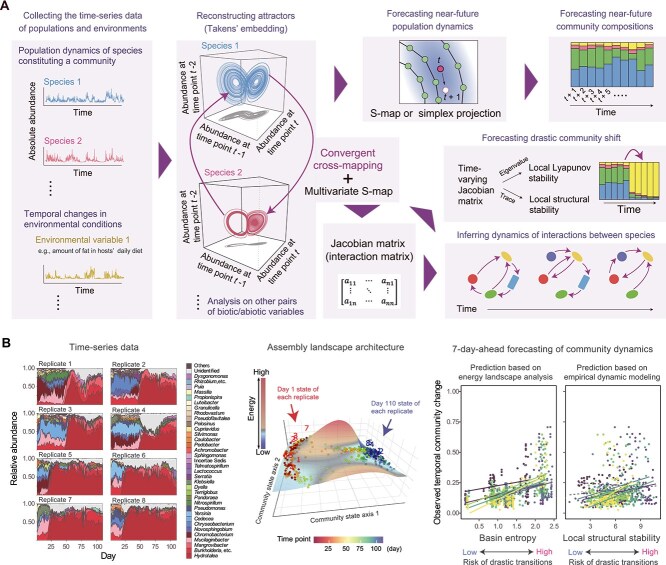
Tracking complex dynamics. (A) Empirical dynamic modeling. A statistical framework assuming complex forms of attractors (attractors with noninteger fractal dimensions) reconstructed from nonlinear (chaotic) time-series data offers an approach for interpreting time-series microbiome data. In empirical dynamic modeling, for example, the attractor of each species’ population dynamics is reconstructed based on the Takens’ embedding theorem [90]. Once the reference databases of attractors are obtained, near-future states of the microbial populations and communities [36] can be forecasted with the aid of simplex projection [87] or S-map [88]. In addition, after applying the convergent cross-mapping analyses for detecting causality between input variables [86], we can examine how the direction and strength of species interactions may change through time [84, 91, 92]. The Jacobian matrices representing interspecific interactions can be used to quantify the instantaneous local contraction/expansion rate around the observed state based on the dominant eigenvalues of the time-varying Jacobian (local Lyapunov stability) [84]. Likewise, the trace (diagonal sum) of the time-varying Jacobian matrix represents the system’s sensitivity/robustness to environmental (or parameter) disturbances at each time point (local structural stability) [93]. Not only the population dynamics of community members but also the time-series data of background environments can be included as state variables in empirical dynamic modeling. Intriguingly, the Jacobian-based framework can be extended to analyses of regulatory networks of metabolites [121] because the same principles of dynamical systems apply [122]. (B) Forecasting community changes. In a laboratory experiment using a soil-derived microbiome [36], the dynamics of eight replicate communities were tracked every 24 h for 110 days (left). For each replicate community, a “reference database” of state space was constructed based on either energy landscape analysis (middle) or empirical dynamic modeling (A) using data from the remaining seven replicates. The magnitude of structural shifts in community composition between the current and seven-day-ahead time points was then forecasted based on basin entropy (Fig. 5A) and local structural stability (A), respectively (right; colors indicate regression lines for each replicate). Reproduced from a previous study [36].

In line with energy landscape analysis and ecological dynamic regime analysis, which are inspired by the classic stability landscape concept, alternative statistical approaches for analyzing time-series microbiome datasets have recently become available. Empirical dynamic modeling [84–88], for example, enables the description of ecological community dynamics by allowing for complex attractor structures. The framework stemming from nonlinear mathematics provides a suite of quantitative approaches for understanding and forecasting temporal dynamics (Fig. 6A), and it is applicable to both microbial and macro-organismal systems [36, 84, 86, 89].

Grounded in Taken’s embedding theorem of attractor reconstruction from time-series data [86, 90], empirical dynamic modeling enables the prediction of near-future states of microbial populations and communities [36] using simplex projection [87] and S-map (sequential locally weighted global linear maps) [88] methods. In addition, through the forecasting of a target species’ population dynamics with the information of the attractors reconstructed from other species’ time-series data, causality within the system (i.e. the direction of species interactions) can be explored using the statistical approach called “convergent cross-mapping” [86]. The mathematical framework further provides a way for estimating the strength of species interactions, yielding a Jacobian matrix (interaction matrix) at each time point [84, 91–93]. The inferred time-varying Jacobian matrices can be used to calculate “local Lyapunov stability” and “local structural stability,” which represent the risk of drastic community structural shifts [84, 93]. An important property of empirical dynamic modeling is that not only biotic state variables (i.e. population densities of community members) but also environmental variables (e.g. nutritional inputs to host animals/plants) can be included in the mathematical framework [86]. Causality among those biotic/abiotic variables can be explored based on a unified theoretical framework designed to exclude indirect (spurious) effects among variables [94]. Although continual efforts are required to improve the accuracy of the attractor reconstruction from noise-containing data [95], empirical dynamic modeling will become an alternative useful tool for describing the trajectories of microbiome processes [36, 85].

## Applying multiple statistical frameworks

When we apply the emerging approaches outlined in this review, the assumptions of each statistical model need to be kept in mind. Energy landscape analysis assumes that data points are distributed around the basin bottoms due to stochastic deviations from attractors [60, 61, 63, 69] (e.g. equilibria or limit cycles; Box 2). By contrast, empirical dynamic modeling has been developed for describing nonlinear (chaotic) behaviors of systems: hence, it assumes essentially deterministic processes around complex forms of attractors (i.e. attractors with noninteger fractal dimensions [84, 86, 91]). In principle, models offer ways by which we formulate natural phenomena. Therefore, multiple modeling frameworks are applicable in quantitatively explaining observed phenomena. In fact, when the two alternative modeling approaches were applied to the same time-series data obtained from a microbiome experiment, both of them provided significant inferences about the risk of abrupt microbiome changes [36] (Fig. 6B).

Therefore, it is premature to conclude which statistical approaches are generally suitable for time-series microbiome analyses. In terms of forecasting, the best statistical approaches may differ depending on the types of microbial ecosystems, the time scale of microbiological phenomena, and data-collection methodologies. For example, because the empirical dynamic modeling approach assumes purely deterministic processes [86], energy landscape analysis and the ecological dynamic regime framework may be more suited to the systems in which stochastic processes play major roles in community assembly [47, 60]. In principle, different modeling approaches shed light on the same phenomena from different perspectives, providing complementary knowledge of the principles governing drastic events in microbiome dynamics.

It is also important to acknowledge that the alternative statistical approaches differ in the criteria for preparing input data. Whereas the statistical-physics-based analysis (energy landscape analysis) is readily applicable to existing datasets of various microbiome projects, the ecological dynamic regime framework uses time series of at least two time points to characterize community assembly [47]. The nonlinear-mathematics-based approach (empirical dynamic modeling) has more stringent criteria for preparing time-series data. In theory, reliable inference of attractors requires time-series data spanning several multiples of the characteristic return time of the target system [96]. The temporal resolution of observation (i.e. the sampling interval) is another critical consideration in the application of the mathematical approach [96], especially when targeting systems with drastic transitions or long transient dynamics. In this context, studies employing daily sampling designs have successfully detected abrupt changes in community structure in studies on human vaginal microbiomes or fish-associated (aquaculture tank water) microbiomes [19, 97]. By contrast, when the objective is to resolve fine-scale temporal phenomena, such as microbiome dynamics driven by the circadian rhythms of host animals or plants [98], much shorter sampling intervals (e.g. hourly sampling) are required. Provided that a dataset contains samples spanning the entire state space of the target dynamics, the time-series statistical framework is applicable to both systems exhibiting continual change and those undergoing abrupt transitions [36]. Another important aspect in applying empirical dynamic modeling is that it requires “absolute” abundance data for reconstructing attractors based on the population dynamics (increase/decrease) of individual species within a target community [86, 91, 92]. From the time-series dynamics perspective, microbiome data expressed in relative-abundance formats therefore lack essential information unless supplemented by quantitative PCR-based workflows [99]. However, “quantitative amplicon sequencing,” which uses standard DNA with defined concentrations to calibrate 16S rRNA gene copy numbers in template DNA aliquots, now enables the acquisition of absolute-abundance time-series data [36, 92, 97], thereby allowing the application of empirical dynamic modeling to microbial ecosystems [36, 92].

Alongside the above-mentioned methods, a variety of mathematical approaches have recently been developed for interpreting seemingly complex microbiome assembly as low-dimensional phenomena [43, 100, 101]. The applications of multiple statistical frameworks to diverse systems will reorganize our understanding of the controllability and predictability of microbiome processes. If statistical approaches with different assumptions vary in their ability to explain or predict ecological dynamics, the fact *per se* will not only accelerate feedback between theoretical and empirical studies but also highlight fundamental properties unique to each microbial system.

## Challenges ahead

Further interdisciplinary integration is necessary for the fundamental understanding of the relationship among the structure, stability, and functions of microbial communities and ecosystems. Updates in the mathematical approaches for describing the behavior of high-dimensional systems will help us determine whether drastic community transitions observed in microbiome time-series data should be interpreted in the conceptual framework of transient states (Fig. 1D and E) or that of assembly landscape shifts along environmental gradients (Fig. 4). In parallel with *in silico* methodological improvements, gaining metadata for systematically characterizing the inferred basins is an essential task (Fig. 1B). The databases of the health/disease status of host individuals are of particular importance [7, 15]. In addition, transcriptomics [102] and phenotyping technologies [103, 104] enable high-throughput survey of morphological and physiological properties of host animals and plants. Likewise, systems biology studies based on metagenomics [105, 106], metabolomics [107, 108], and “panomics” [109] (Box 1) allow the evaluation of the physiological interplay between microbiomes and their hosts [110, 111]. Furthermore, cutting-edge technologies for imaging >1000 microbial taxa with fluorescent probes [112] and those for estimating the replication rates of respective bacteria constituting a community [113] will shed new light on the functional roles of microbiomes.

Gaining systematic insights into microbial interaction networks is an important direction of research as well. By estimating the metabolic capabilities of respective species based on their gene repertoires [114, 115], for example, we can infer the extent to which community members compete for the same resources [116]. Such insights will be helpful for anticipating community collapse triggered by severe overlap of resource demands between constituent species [117] (i.e. competitive exclusion between species with similar metabolic profiles [118, 119]). By contrast, communities with minimal levels of competition would contain empty niches: hence, such microbiomes would be susceptible to invasions (or contaminations) of alien species [116]. Metagenomic information can also be used to estimate possible flows of metabolites between species [120] or regulatory interactions between metabolites [121, 122]. Within the suggested networks, species or metabolites located at the key topological positions (potential regulatory checkpoints) may be designated as candidate drivers of whole-ecosystem-scale processes [121]. It remains to be experimentally examined how the manipulation (elimination or genetic modifications) of such candidate keystone species [123] or “keystone metabolites” [124, 125] can entail the entire architecture of assembly landscapes.

Another important challenge is to develop algorithms for finding the optimal routes of community changes within the multi-dimensional space of environmental variables. Manipulations of single biotic/abiotic factors do not necessarily entail desirable shifts in community structure and related functions. To find the optimal sequences of assembly landscape changes for realizing favorable community shifts, the order and magnitude of controlling multiple environmental variables need to be explored. In this respect, algorithms for exploring potential routes of sequential chemical reactions [126] or cell developmental trajectories [64] will give essential insights.

In deepening the bird’s-eye knowledge of microbiome structure and functions on dynamically changing assembly landscapes, it is essential to integrate the top–down data-driven approach with bottom-up experimental studies. High-throughput experiments of synthetic communities [15, 25, 77], for example, will accelerate our understanding of how assembly landscape architecture changes depending on environmental conditions. In particular, synthetic community experiments based on microfluidics [127] enable rapid profiling of microbiome-scale functions, providing opportunities for screening stable and highly functional compositions of microbial species. Ultimately, such selected sets of “core” microbes may be embedded into real microbial ecosystems with full complexity, working as built-in stabilizers or promoting shifts into targeted microbiome states with favorable functions [4, 12, 14]. Strategic design and control of assembly landscapes will consolidate fundamental practical bases for deploying demanded microbiome functions for diverse medial, agricultural, and industrial purposes.

## Data Availability

The following figures were drawn based on the data publicized in the previous studies: Fig. 4, references 51 and 74; Fig. 5D, reference 82; and Fig. 6B, reference 36.

## Code availability

The program codes used for making Figs 4–6 are available on the Zenodo repository (10.5281/zenodo.19244425).
